# Beyond yellow: discovery and genetic dissection of an apricot petal color in *Brassica juncea* L

**DOI:** 10.3389/fpls.2026.1767871

**Published:** 2026-02-02

**Authors:** Ri hui Li, Xiao xue Zhang, Kai wen Yuan, Yan li Zhao, Cheng tao Quan, Kai xuan Wang, Rong zhan Guan, He jun Lu, Jia shun Miao, Dong qing Zhang

**Affiliations:** 1 Xianghu Laboratory, Institute of Biological Seed Industry, Hangzhou, China; 2 State Key Laboratory of Crop Genetics & Germplasm Enhancement and Utilization, Jiangsu Collaborative Innovation Center for Modern Crop Production, Nanjing Agricultural University, Nanjing, China

**Keywords:** anthocyanins, *Brassica juncea*, BSA-seq, flower color, multi-omics, *PAP2*

## Abstract

*Brassica juncea* is an important oilseed and vegetable crop whose flowers are typically yellow, largely owing to carotenoid pigmentation. Here, we report a novel, heritable apricot-flowered variant designated ‘Caijie,’ from a wild *B. juncea* accession. Metabolomic profiling revealed that the distinctive petal coloration was primarily attributable to anthocyanin accumulation. Genetic mapping via bulked segregant analysis (BSA) mapped the apricot-flowered trait to a single dominant locus within a 9.76-Mb interval on chromosome B03. Among the 1,406 annotated genes in this region, Production of Anthocyanin Pigment 2 (*BjB03.PAP2*), which encodes an R2R3-MYB transcription factor, emerged as the most likely candidate gene. Consistent with this, transcriptomic analysis revealed coordinated upregulation of multiple structural genes involved in the anthocyanin biosynthesis pathway in the apricot-flowered variant. Further sequence analysis revealed a (TC)_n_ dinucleotide repeat polymorphism in the promoter of *BjB03.PAP2*, representing a structural variation that is likely responsible for enhanced transcriptional activity and subsequent anthocyanin production in petals. This study unveils a previously unrecognized genetic mechanism underlying flower color variation in *B. juncea*, offering new insights into the evolution of floral pigmentation and a valuable genetic resource for breeding ornamental Brassica crops.

## 1 Introduction

*Brassica juncea* (L.) Czern & Coss is an allopolyploid species (AABB, 2n = 36) that originated from hybridization between the diploid progenitors of *B. rapa* (AA, 2n = 20) and *B. nigra* (BB, 2n = 16) (Kang et al., 2021). This species formed approximately 8,000–14,000 years ago and was domesticated around 3000 BCE (Kang et al., 2021). It has since developed into an important crop with diverse uses, and the fresh roots, stems, and leaves are consumed as nutritious vegetables, whereas the seeds serve both as a major source of edible vegetable oil and as a raw material for condiments, such as mustard (Yang et al., 2016; Kang et al., 2021; Zhang et al., 2025). The rich genetic reservoir of *B. juncea*, harboring advantageous traits such as disease resistance, abiotic stress tolerance, and diverse morphotypes, offers a promising germplasm for the breeding and improvement of Brassica crops. Despite its importance as a genetic reservoir, fundamental biological research on *B. juncea*, particularly on the genetic control of key traits, has lagged behind that of its close relatives, including *B. napus* (AACC, 2n = 38). The latter is also an allotetraploid species derived from the hybridization between *B. rapa* (AA, 2n = 20) and *B. oleracea* (CC, 2n = 18) and serves as a crucial and primary winter oilseed crop in China.

As a conspicuous morphological trait, petal color plays a significant role in plant taxonomy and evolutionary studies and is a key characteristic for attracting pollinators (Trunschke et al., 2021; Liang et al., 2023; Kellenberger and Glover, 2023). Additionally, its ornamental value drives the growth of agricultural tourism. Brassicaceae plants exhibit a rich diversity of petal colors, which arise from the accumulation of various pigments, such as carotenoids and anthocyanins (Nikolov, 2019). Carotenoid pigmentation is modulated by the complex interplay between synthesis, degradation, and storage mechanisms. Mutations in core carotenoid biosynthetic genes, such as *PDS*, *CRTISO*, and *ZEP*, can alter carotenoid content and composition, leading to pale yellow, white, or orange petals in *B. napus* (Liu et al., 2020; Zhao et al., 2021; Li et al., 2022). Furthermore, plastid-localized Carotenoid Cleavage Dioxygenases 4 (*CCD4*) can degrade pigments and produce white petals (Zhang et al., 2015a; Han et al., 2019). In addition to biosynthesis and degradation, white or pale yellow phenotypes can arise from disruptions in carotenoid storage pathways. This occurs through mutations in genes such as Xanthophyll Esterases (*XES*), which are involved in carotenoid esterification, and *FBN1b*, a fibrillin gene essential for plastoglobule formation that prevents massive carotenoid accumulation (Li et al., 2023).

In recent years, colored rapeseed flowers, which are characterized by anthocyanin accumulation, have attracted considerable interest. By using distant hybridization, researchers have successfully introduced critical genes or alleles that activate the anthocyanin biosynthesis pathway in the petals, thereby enriching the flower color palette. A red-flowered *B. napus* line was developed via hybridization with the purple-flowered crucifer *Orychophragmus violaceus*, resulting in a stable disomic addition line (Fu et al., 2018). Similarly, crossing purple-flowered radish (Raphanus) provides an alternative route for introducing key alleles that activate the anthocyanin pathway in *B. napus* (Xiao et al., 2022). Subsequent crosses of these distant hybrid derivatives with the dominant white or yellow flowers of *B. napus* resulted in a broad segregation of petal colors in the progeny, encompassing yellow, white, red, pink, and pale purple hues (Xiao et al., 2022; Ye et al., 2022). This diversity is produced by varying the types and proportions of carotenoids and anthocyanins. Subsequently, a key candidate gene from these materials was cloned and identified as the R2R3-MYB transcription factor Production of Anthocyanin Pigment 2 (*PAP2*). Transgenic experiments confirmed that *PAP2* activates the expression of anthocyanin biosynthesis genes (Fu et al., 2018; Ye et al., 2022). This activation follows the classic MYB-bHLH-WD40 (MBW) transcriptional complex regulatory model in which PAP2 (an R2R3-MYB protein) interacts with a bHLH partner, such as TRANSPARENT TESTA 8 (TT8), and a WD-repeat protein, such as TRANSPARENT TESTA GLABRA1 (TTG1), to form a functional complex (Xu et al., 2015). The MBW complex subsequently upregulates key structural genes in the anthocyanin pathway, including dihydroflavonol 4-reductase (*DFR*), anthocyanidin synthase (*ANS*), flavonoid 3-O-glycosyltransferase (*UFGT*), methyltransferase (*MT*), and glutathione S-transferases (*GST*), which ultimately leads to substantial anthocyanin accumulation in rapeseed petals (Ye et al., 2022). Furthermore, bioengineering has expanded the *B. napus* pigment palette by introducing non-native pigments, such as betalains, alongside endogenous carotenoids and anthocyanins, thereby enabling the rational design of diverse novel flower colors (Zheng et al., 2025). However, most Brassica crops predominantly display yellow petals, a hallmark trait of the genus that is primarily attributed to carotenoid-based pigmentation (Li et al., 2024). Consequently, the potential of rapeseed as an ornamental crop is limited by its lack of floral color diversity and insufficient genetic resources for color manipulation (Xiao et al., 2022).

Here, we report the identification of a striking, stably inherited, apricot-flowered variant from wild *B. juncea* germplasm collected from the coastal region of Wenzhou, Zhejiang Province, China. This unexpected phenotype challenges the conventional understanding of flower color in this species and suggests the involvement of a previously undiscovered genetic regulatory pathway. Metabolomic profiling revealed that the apricot-flowered phenotype is primarily attributed to anthocyanin accumulation. Genetic analysis indicated that the trait is governed by a single dominant locus, which was preliminarily mapped to a 9.76 Mb interval on chromosome B03. Among the candidate genes within this region, *BjB03.PAP2* emerged as the most promising candidate, and sequence analysis revealed a structural variation consisting of polymorphic (TC)_n_ dinucleotide repeats in the promoter region of *BjB03.PAP2*, which is likely to alter its transcriptional activity and consequently lead to enhanced anthocyanin accumulation in the petals. Deciphering the genetic basis of this unique trait not only holds theoretical importance for understanding the evolution of pigment biosynthesis pathways in Brassica species but also provides valuable genetic resources for molecular breeding aimed at improving ornamental value and agronomic traits.

## 2 Materials and methods

### 2.1 Plant materials and growth conditions

The white-flowered inbred line ‘JG800’ (WP) of *B. juncea* L., which exhibits a stable white petal phenotype, and ‘Caijie’ (CJ), a natural variant characterized by its apricot-flowered trait, were used as the parental material in this study. The CJ germplasm was originally collected from the coastal area of Wenzhou, Zhejiang Province, China, and self-pollinated for at least three generations under greenhouse conditions to ensure phenotypic stability. F_1_ hybrids were generated by crossing WP with CJ, and the F_2_ population was subsequently obtained by self-pollinating the F_1_ plants. All materials were cultivated in a greenhouse set to 23°C with a 16-h light/8-h dark photoperiod for phenotypic evaluation.

### 2.2 Phenotypic scoring and genetic analysis

Petal color in the F_2_ population was scored based on the presence or absence of visible anthocyanin pigmentation. Petals exhibiting apricot or pink coloration were classified as anthocyanin-pigmented, whereas yellow and white petals were classified as anthocyanin-deficient. Segregation ratios in the F_2_ population were tested for goodness-of-fit to expected Mendelian ratios using the chi-square (χ²) test. The chi-square value was calculated using the following formula:


$${\text{χ}}^{2}=\sum \left[\;({\text{O}}_{i}-{\text{E}}_{i}{)}^{2}/{\text{E}}_{i}\right]$$

where O_i_ is the observed number of individuals in phenotype class ‘I,’ E_i_ is the expected number of individuals in that class based on the genetic hypothesis, and Σ denotes the summation over all phenotypic classes. The degrees of freedom (df) were calculated as ‘n’ − 1, where ‘n’ is the number of phenotypic categories. A *P*-value > 0.05 was used as the threshold to indicate that the observed data did not deviate significantly from the expected genetic ratio, thus supporting the proposed genetic model.

### 2.3 Anthocyanin profiling and RNA-seq analysis

The flower buds and fully opened flowers of the WP and CJ lines were collected at four distinct developmental stages: small buds (stage S1), medium buds (stage S2), large buds (stage S3), and fully opened flowers (stage S4). All samples were immediately frozen in liquid nitrogen and stored at −80°C for subsequent anthocyanin and transcriptome analyses. Anthocyanin profiles were analyzed using ultra-performance liquid chromatography–tandem mass spectrometry (UPLC–MS/MS). Extraction, detection, and quantification of anthocyanins were performed by METWARE Biotechnology Co., Ltd. (Wuhan, China; http://www.metware.cn/) according to standard protocols. For the transcriptome analysis, total RNA was extracted from the same set of samples. Library construction and RNA sequencing were performed by METWARE Biotechnology Co., Ltd., using the Illumina platform, followed by bioinformatics analysis of the generated data.

### 2.4 Bulked segregant analysis sequencing

Two bulked DNA samples were constructed from the F_2_ population based on flower phenotypes. The “Anth-bulk” comprised equal amounts of genomic DNA from 30 apricot- and 2 pink-flowered plants, reflecting the expected 15:1 segregation ratio. Similarly, the “NonAnth-bulk” was constructed from 30 yellow- and 2 white-flowered plants. Genomic DNA from two parental lines (WP_77 and CJ_72) was also included, with one individual per parent used as a reference. Genomic DNA was extracted from the fresh leaf tissue of all selected individuals using MolPure Plant DNA Kit (Yeasen Biotechnology, Cat. No. 18800ES50). Four DNA sequencing libraries (two parental and two bulk libraries) were constructed. Paired-end sequencing was performed on an Illumina NovaSeq Plus platform by Novogene Corporation (Beijing, China).

BSA-seq was performed using the *Brassica juncea* reference genome AU213 (http://brassicadb.cn/) (Yang et al., 2021; Chen et al., 2022). Initially, SNPs and InDels identified against this reference and passing GATK hard filtering were subjected to a genotype missing-rate filter using VCF tools (max-missing 0.75) (Danecek et al., 2011). This step retained only sites that were successfully genotyped in at least three of the four samples (two parents and two trait bulks). Key information, including chromosome position, alleles, genotype calls, and read depths, was extracted using GATK (McKenna et al., 2010). Subsequent analysis was conducted using the R package easyQTLseq: the maternal line CJ_72 and its high-trait bulk were designated as the high group, whereas the paternal line WP_77 and its low-trait bulk were designated as the low group, with the population type defined as F_2_. Sliding-window statistics (SNP-index, Δ(SNP-index), and Euclidean distance (ED)) were calculated using window sizes of 1 or 2 Mb and a step size of 20 kb (Takagi et al., 2013). Windows containing fewer than 20 informative SNPs were excluded, and 95% and 99% confidence intervals were estimated by simulation under the null hypothesis of no QTL. Finally, genomic windows ranked in the top 1% by absolute |Δ(SNP-index)| values were defined as putative QTL regions.

### 2.5 Reverse transcription and quantitative real-time PCR analysis

Petals (~200 mg) were collected from S3 stage flower buds of F_2_ individuals exhibiting four distinct colors (apricot, pink, yellow, and white) to validate the gene expression patterns. Samples were immediately frozen in liquid nitrogen for subsequent RNA extraction. Total RNA was isolated using Eastep Super Total RNA Extraction Kit (Promega). Genomic DNA was removed during the extraction to obtain high-quality RNA. First-strand cDNA was synthesized from 3 µg of total RNA using RevertAid First Strand cDNA Synthesis Kit (Thermo Scientific, Cat. No. K1622) in a 20 µL reaction volume, following the manufacturer’s instructions. The cDNA was diluted 50-fold with nuclease-free water. qRT-PCR was performed in 10 µL reaction volumes containing 4.6 µL of diluted cDNA template, 0.2 µL each of forward and reverse primers (10 µM), and 5.0 µL of KAPA SYBR FAST Universal qPCR Master Mix (Cat. No. KK4601). Reactions were performed on QuantStudio 3 Real-Time PCR System (Thermo Fisher Scientific). All primer sequences used in this study are listed in **Supplementary Table 1**.

## 3 Results

### 3.1 Single dominant locus control the apricot-flowered trait

**Figure 1:**
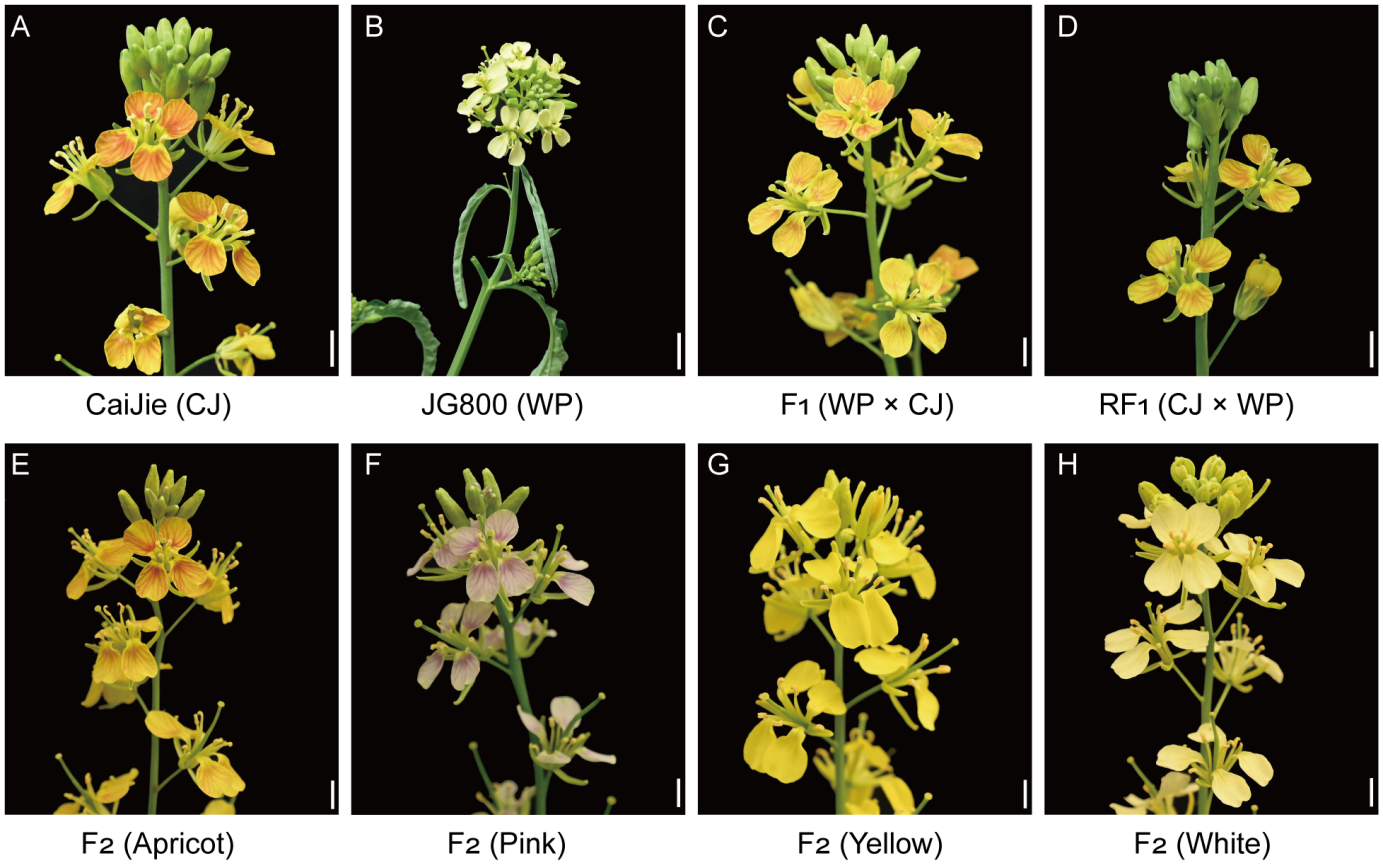
Flower color phenotypes of the parental lines, F_1_, RF_1_, and F_2_ populations in *B. juncea*. **(A, B)** Inflorescences of the apricot- and white-flowered parental individuals. **(C, D)** Inflorescences of the apricot-flowered F_1_ and RF_1_ individuals. **(E–H)** Four distinct flower color phenotypes segregated in the F_2_ population. Scale bar: 5 mm.

The CJ germplasm is a natural variant possessing the apricot-flowered (*APF*) trait, which has been stabilized through successive selfing generations (**Figure 1A**). The WP was selected as the parent for hybridization to introduce a novel color variation into its progeny (**Figure 1B**). A previous study established that the white petal phenotype of WP results from simultaneous loss-of-function mutations in the functionally redundant xanthophyll esterase genes *BjA02.XES* and *BjB04.XES* disrupt carotenoid storage (Li et al., 2023). We crossed WP with the apricot-flowered parent CJ to generate F_1_ and F_2_ populations to determine the inheritance of the *APF* locus. The uniform apricot-flowered phenotype observed in both the F_1_ and reciprocal F_1_ (RF_1_) populations demonstrates that this trait is controlled by a dominant nuclear gene at the *APF* locus, with no detectable cytoplasmic effects (**Figures 1C, D**).

In the F_2_ population, 1,187 individuals were obtained and segregated into four distinct phenotypic classes: apricot, pink, yellow, and white (**Figures 1E–H**). Genetic analysis of the F_2_ population confirmed that the *APF* locus is the single dominant gene controlling a novel pigmentation pathway. Two observations supported this conclusion. First, the segregation of apricot + pink versus yellow + white petals fits a 3:1 ratio (χ² = 1.11, *P* = 0.29), demonstrating monogenic dominant inheritance (**Table 1**). Second, when considering carotenoid accumulation independent of the *APF* locus, the segregation of carotenoid-pigmented (apricot and yellow) versus non-pigmented (pink and white) petals fitted a 15:1 ratio (**Table 1**). This indicates that the presence of carotenoids is controlled by two redundant genes, *BjA02.XES* and *BjB04.XES* (Li et al., 2023). Critically, the two pathways are genetically independent, as the overall segregation perfectly fits a 45:3:15:1 dihybrid ratio (χ² = 1.97, *P* = 0.58), confirming that no epistasis exists between the *APF*-mediated and carotenoid pathways (**Table 1**). In summary, we established that the *APF* locus is a single dominant gene controlling a novel pigmentation pathway that is genetically independent of the carotenoid biosynthesis pathway governed by redundant *XES* genes.

**Table 1:** Phenotypic segregation for petal color in the F_1_ and F_2_ populations.

Generation	Total plants	Apricot	Pink	Yellow	White	Expected ratio	χ² value	*P*-value
CJ	3	3	–	–	–	–	–	–
WP	3	–	–	–	3	–	–	–
F_1_ (WP × CJ)	150	150	0	0	0	1:0 (A+P: Y+W)^1^	–	–
RF_1_ (CJ × WP)	127	127	0	0	0	1:0 (A+P: Y+W)	–	–
F_2_	1187	856	50	264	17	3:1 (A+P: Y+W) 15:1 (A+Y: P+W)	^2^1.11(A+P: Y+W) ^2^0.73 (A+Y: P+W)	*P* = 0.29 > 0.05 *P* = 0.39 > 0.05
						45:3:15:1 (A: P: Y: W)	^3^1.97 (A: P: Y: W)	*P* = 0.58 > 0.05

^1^A, Apricot; P, Pink; Y, Yellow; W, White; ^2^χ² = 1.11 or 0.73< χ² 0.05 = 3.84, *P* > 0.05; ^3^χ² = 1.97< χ² 0.05 = 7.82, *P* > 0.05, indicates no significant difference between the observed and expected ratios.

### 3.2 Anthocyanins are associated with the apricot-flowered phenotype

**Figure 2:**
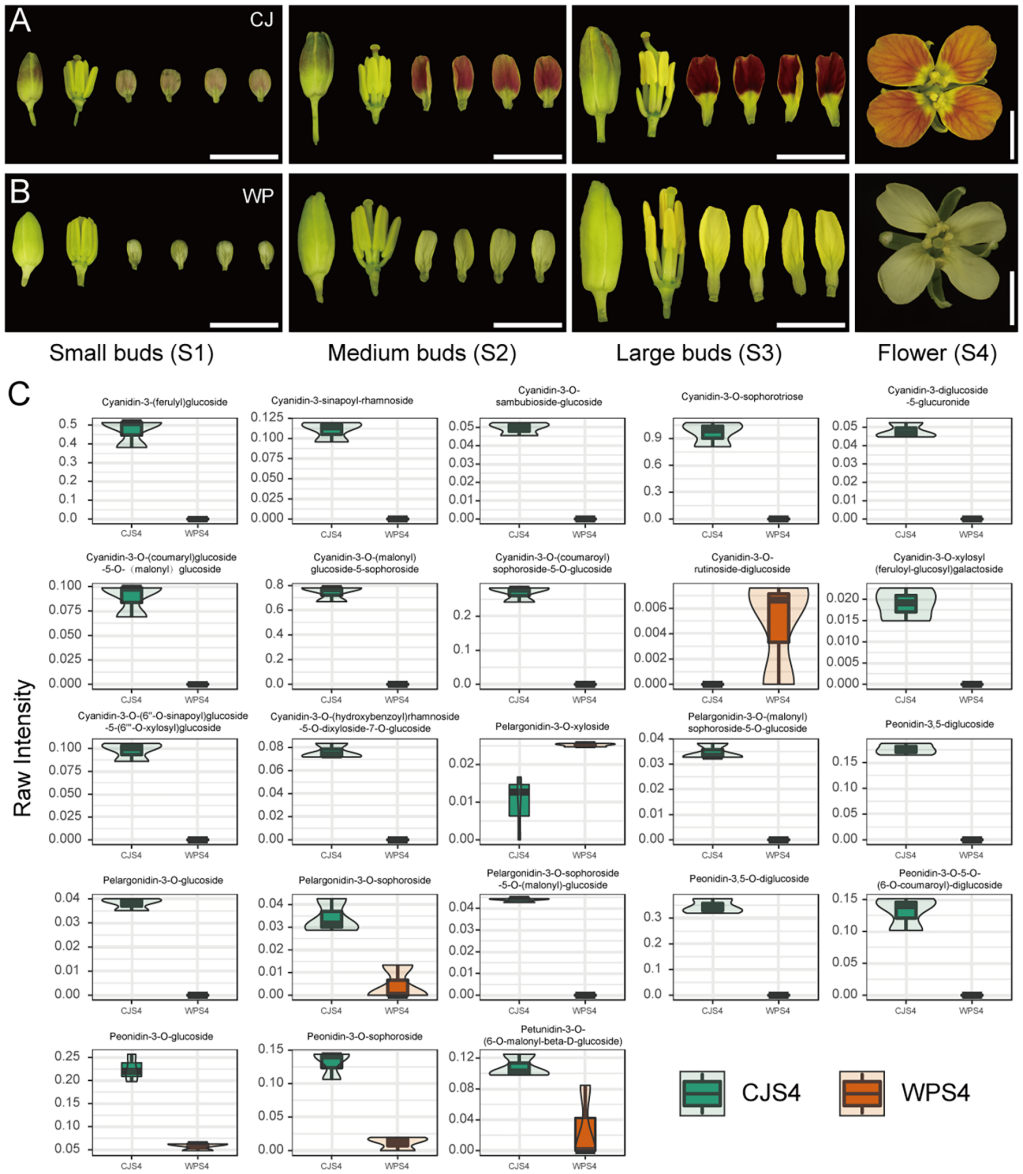
Anthocyanin accumulation across developmental stages and violin plots of differential accumulated anthocyanins. **(A, B)** Representative images showing anthocyanin pigmentation in the sepals, petals, and anthers of the two parental lines, CJ and WP, across four floral bud developmental stages (S1–S4). Scale bar: 5 mm. **(C)** Violin plots comparing the raw intensities of anthocyanins that were differentially accumulated between CJ and WP flowers at the S4 stage.

The apricot petal phenotype and visible red pigmentation are typically attributed to anthocyanin accumulation. For instance, the expression of *BnaA07.PAP2* induces anthocyanin deposition in both the petals and anthers of *B. napus* (Ye et al., 2022). To ascertain the pigment composition of the petals, we conducted detailed observations of petal tissues at different developmental stages from the parental lines CJ and WP, as well as their hybrid progeny. Petal and calyx observations confirmed developmentally intensified red pigmentation in CJ and its apricot- or pink-flowered progeny but not in WP or its yellow and white descendants (**Figures 2A, B**; **Supplementary Figure 1**). The absence of red pigmentation in the anthers across all materials indicated that the *APF* locus acts in a spatially distinct manner and may have a different genetic basis than *BnaA07.PAP2* (**Figure 2A, B**; **Supplementary Figure 1**).

We performed targeted metabolomic analysis using UPLC–MS/MS on the bud and flower samples of CJ and WP collected at four key developmental stages (S1–S4) to further quantify the composition and concentration of anthocyanins. We profiled 53 anthocyanins and their derivatives. The results revealed a consistent pattern of differential accumulation in CJ buds compared with that in WP during development. Particularly, the number of anthocyanins showing upregulated accumulation in CJ increased from 10 in S1 to 14 in S2, 16 in S3, and 21 in S4 (**Figure 2C**; **Supplementary Table 2**). Concurrently, the numbers of downregulated anthocyanins were 1, 3, 4, and 2 at S1 through S4, respectively, indicating that upregulation was the predominant pattern (**Figure 2C**; **Supplementary Table 2**). Targeted metabolomics revealed that the apricot flower phenotype is associated with a developmentally enhanced anthocyanin profile.

### 3.3 BSA-based mapping of the candidate gene controlling anthocyanin-based flower color

BSA-seq was performed to identify genomic regions contributing to apricot-flowered development. The two parental lines, along with two bulked pools, Anth-bulk and NonAnth-bulk, were subjected to whole-genome resequencing. NGS generated high-quality paired-end reads for all four samples. After quality filtering, >95% of reads from each sample were retained. Mapping rates to the *B. juncea* AU213 reference genome exceeded 99.13%, and duplication rates ranged from 14.63% to 18.62% (**Supplementary Table 3**). The mean sequencing depths for the parents were approximately 8–9×, whereas the two bulks exhibited depths of 31× and 33× (**Supplementary Figure 2A**). Joint genotyping identified ~5.39 million high-quality SNPs and ~1.57 million high-quality InDels across the parental lines and bulks. After applying hard filtering and missing-rate thresholds, ~3.80 million SNPs and ~1.07 million InDels were retained for BSA. The variants were uniformly distributed across the 18 chromosomes of the AA and BB subgenomes (**Supplementary Figure 2B**).

**Figure 3:**
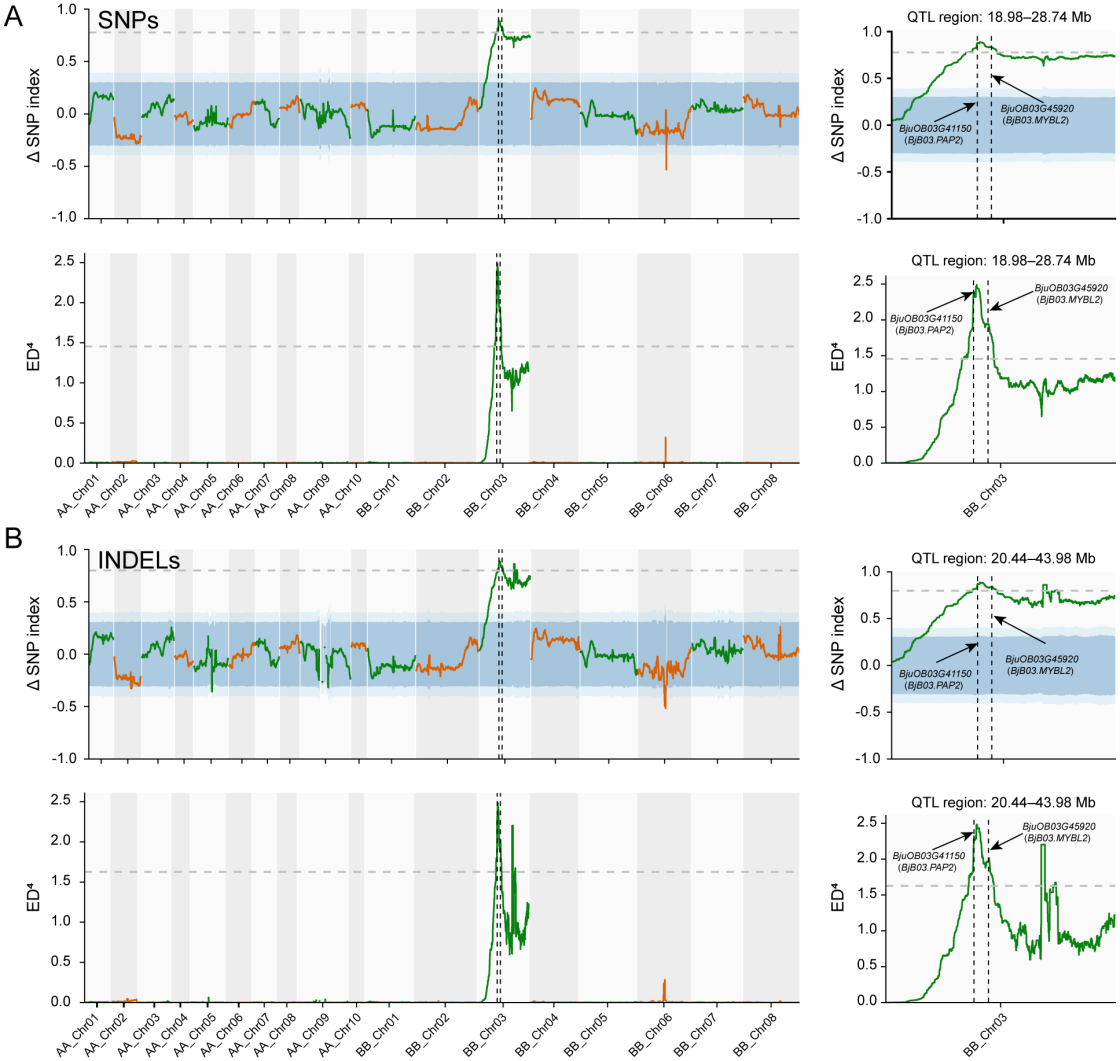
Chromosomal distribution of SNP- and InDel-association values from BSA-seq analysis. **(A)** Distribution of SNP-index values. The peak on chromosome B03 (18.98–28.74 Mb) delineates the candidate region harboring the *APF* locus. **(B)** Distribution of InDel-index values. Black dashed lines indicate the genomic positions of previously characterized genes involved in anthocyanin synthesis or regulation within the candidate region.

For each segregating site, the SNP-index (allele frequency of the alternative allele) was calculated for both the Anth-bulk and NonAnth-bulk. The Δ(SNP-index) was computed as the difference between bulk indices. Sliding-window analysis (window size = 2 Mb, step size = 20 kb) revealed pronounced genomic regions exhibiting strong deviations in Δ(SNP-index), suggesting linkage to *APF* loci (**Figures 3A, B**). A major QTL was detected on chromosome BB_Chr03, where ΔSNP-index values showed a strong deviation from expected Mendelian segregation, and consistently exceeded the 99% confidence interval threshold across an approximately 47.41 Mb region (10.38–57.79 Mb). The peak SNP was located at 23.48 Mb, where the ΔSNP-index reached its maximum (0.89). Using the top 1% ΔSNP-index threshold, a large continuous interval (Chr03: 18.98–28.74 Mb) was identified, corresponding to the major QTL for flower color. No other chromosomes contained extended high-ranking windows, indicating a single major-effect locus (**Figures 3A, B**). To complement Δ(SNP-index), ED between bulk allele-depth vectors was computed across all variants. Peaks in ED coincided with major Δ(SNP-index) peaks, further supporting candidate intervals (**Figures 3A, B**).

Collectively, both Δ(SNP-index) and ED analyses convergently identified strong signals on chromosome BB_Chr03 (18.98–28.74 Mb), representing major-effect genomic regions associated with apricot-flowered phenotype. These intervals span approximately 9.76 Mb and contain 1,406 annotated genes, including transcription factors and pigment biosynthetic genes (*BjuOB03G41150* and *BjuOB03G45920*) with known roles in anthocyanin accumulation (**Figures 3A, B**).

### 3.4 Screening of DEGs associated with the *APF* locus using RNA-seq analysis

**Figure 4:**
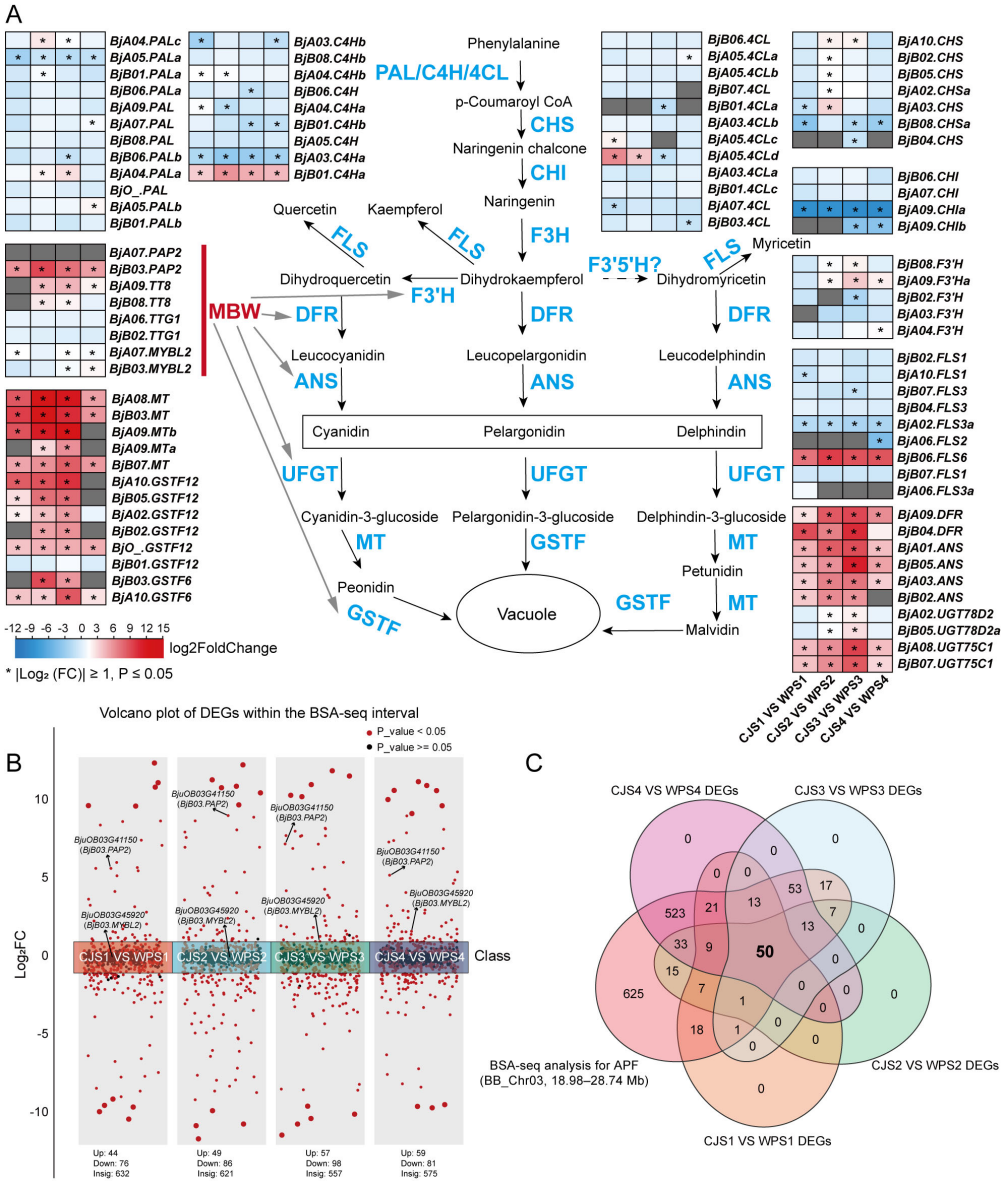
Expression of the anthocyanin biosynthesis pathway expression and candidate gene analysis. **(A)** Heatmap showing DEGs (log_2_FC) in the anthocyanin biosynthesis pathway across flower developmental stages (S1–S4). Gene IDs are provided in **Supplementary Table 5**. Gene abbreviations: PAL, phenylalanine ammonia-lyase; C4H, cinnamic acid 4-hydroxylase; 4CL, 4-coumarate-CoA ligase; CHS, chalcone synthase; CHI, chalcone isomerase; F3H, flavanone 3-hydroxylase; F3'H, flavonoid 3'-hydroxylase; F3'5'H, flavonoid 3',5'-hydroxylase; FLS, flavonol synthase. **(B)** Volcano plot of DEGs between CJ and WP within the BSA candidate region. **(C)** Venn plot of DEGs across developmental stages (S1–S4) within the BSA candidate region. The asterisk (*) indicates ∣log2FC∣≥1 with P ≤ 0.05.

RNA-seq was conducted on four developmental stages of flower buds (S1–S4) from the CJ and WP parental lines to refine the candidate gene pool (**Figures 2A, B**). High-quality sequencing was achieved, with 86.38–92.92% of reads per sample mapping to the *B. juncea* genome, and high replicate concordance validated data robustness (**Supplementary Figure 3**; **Supplementary Table 4**). Differential expression analysis was performed through pairwise transcriptome comparisons between the differently colored buds. Following the differential expression analysis, we focused on genes involved in the anthocyanin biosynthesis pathway, given their established role in governing the apricot-flowered phenotype (**Supplementary Table 5**). Early biosynthetic genes (EBGs) of the flavonoid pathway are largely non-differentially expressed. Nonetheless, several EBGs were significantly deregulated in CJ buds: 16 genes (including *BjA04.PALa*, *BjB01.C4Ha*, *BjA05.4CLd*, and *BjA10.CHS*) were upregulated, and a distinct set of 16 genes (including *BjA05.PALa*, *BjA03.C4Ha*, *BjA07.4CL*, *BjB08.CHSa*, and *BjA09.CHIa*) were downregulated (|log_2_ Fold Change| > 1, **Figure 4A**). In contrast to EBGs, most late biosynthetic genes (LBGs) in CJ buds, including key enzymes such as *FLS*, *DFR*, *ANS*, *UFGT*, *MT*, and *GST*, were significantly upregulated across all four developmental stages (**Figure 4A**). Notably, we observed an upregulated expression of genes encoding both positive (*PAP2* and *TT8*) and negative (*MYBL2*) regulatory components of the MBW complex in CJ buds (**Figure 4A**). This suggests that an altered balance within this master regulatory complex may be the key driver of the widespread upregulation of LBGs.

Given that our genetic analysis indicated *APF* is a dominant single-gene locus, we hypothesized that the dominant allele confers enhanced expression. Therefore, we focused on the subsequent screening of genes upregulated in the CJ line. Next, we analyzed the differential expression of 1,406 annotated genes within the *APF* candidate region. Across the four developmental stages, this interval contained 44–59 upregulated and 76–98 downregulated genes (**Figure 4B**). Notably, the anthocyanin-positive regulator *BjB03.PAP2* was upregulated at all stages (**Figure 4B**), whereas its homolog *BjA07.PAP2* was not detected (**Figure 4A**). For the negative regulator *BjB03.MYBL2*, expression did not differ between CJ and WP at stages S1 and S2 but was upregulated in CJ at S3 and S4 (**Figure 4B**). Its homolog *BjA07.MYBL2* was upregulated in CJ at stages S1, S3, and S4 (**Figure 4A**). In line with this genetic model and the fact that differences in anthocyanin accumulation are established from the early stages (S1 and S2), the late and stage-specific upregulation of *BjB03.MYBL2* was excluded as a candidate gene. Finally, to identify high-confidence candidates, we intersected the DEGs from all four stages with genes from the 9.76 Mb BSA-seq interval (**Supplementary Table 6**). This process yielded a refined set of 50 DEGs, comprising 23 upregulated and 27 downregulated genes (**Figure 4C**; **Supplementary Table 7**).

### 3.5 Gene annotations and sequence variants analyses in candidate genes

**Figure 5:**
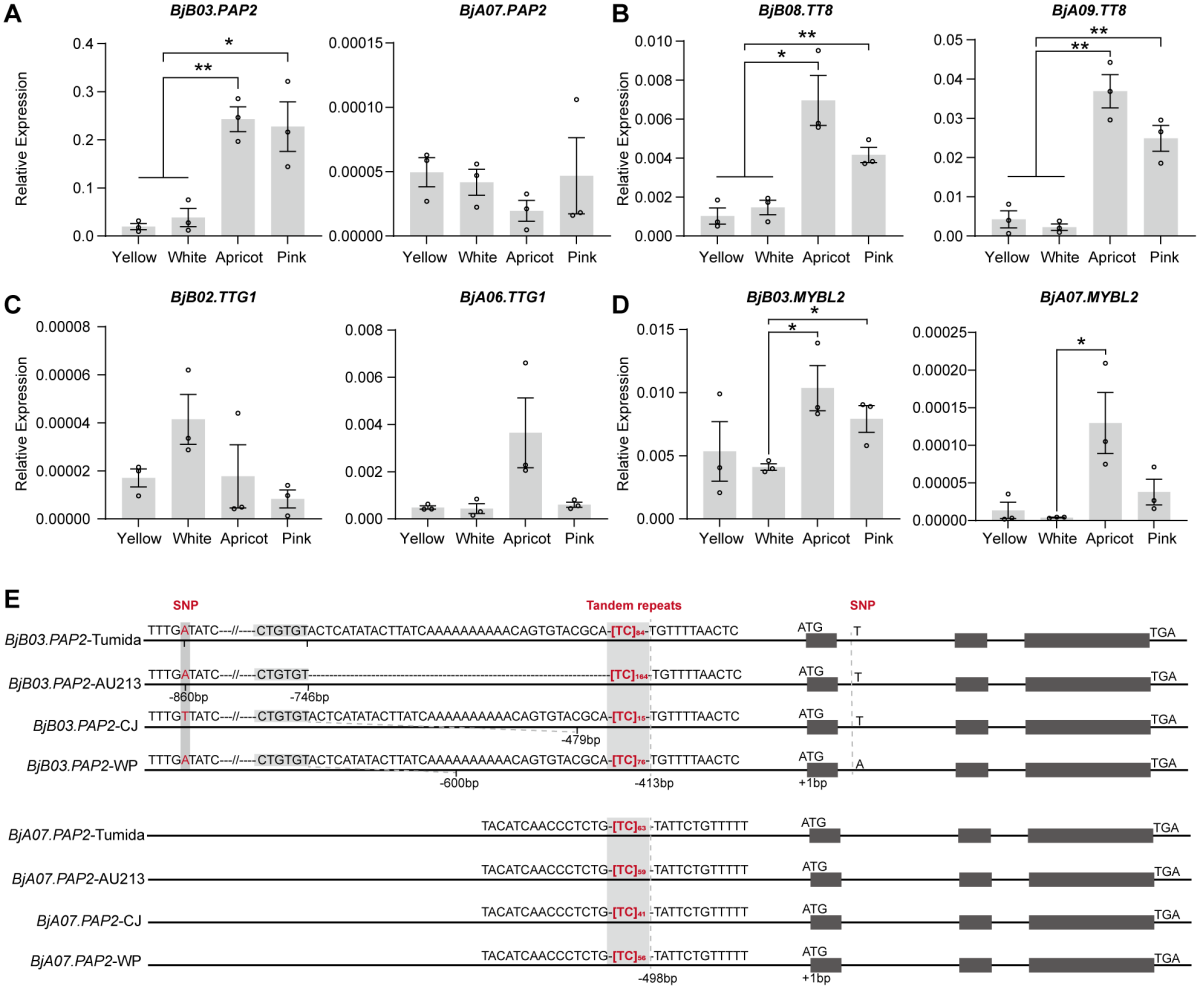
Expression analysis of MBW complex genes and comparative sequencing of the candidate gene *PAP2*. **(A–D)** Relative expression levels of *PAP2*, *TT8*, *TTG1*, *MYBL2*, and their homologs in S3-stage petals of the four flower color types in the F_2_ population. Expression levels were normalized to *ACTIN2* and calculated using the 2^^-^ΔCt^ method. Data are presented as mean ± SE of three biological replicates. Significant differences were determined using a two-sided Student’s *t*-test (*P < 0.05; **P < 0.01). **(E)** Comparative sequence analysis of the candidate gene *BjB03.PAP2* and its homolog *BjA07.PAP2* across two reference genomes (Tumida and AU213) and the parental lines CJ and WP.

We performed a comparative annotation with *Arabidopsis* homologs to functionally characterize the 23 consistently upregulated candidates. This analysis identified *BjuOB03G41150* (*BjB03.PAP2*) as a key candidate due to its known role in anthocyanin regulation, whereas the other 22 genes showed no links to flavonoid/anthocyanin pathways (**Table 2**). We quantified the expression of core MBW complex genes via qPCR in S3 petals of F_2_ plants to validate the transcriptome data. *BjB03.PAP2* expression was significantly higher in anthocyanin-rich apricots and pink petals than in yellow and white petals (**Figure 5A**). Its homolog, *BjA07.PAP2*, showed low and no differential expression across all petal types (**Figure 5A**). Similarly, the positive regulators *BjB08.TT8* and *BjA09.TT8* were significantly upregulated in apricot and pink petals (**Figure 5B**). In contrast, the expression of *BjB02.TTG1* and *BjA06.TTG1* did not differ significantly among petal types (**Figure 5C**). Both negative regulators, namely, *BjB03.MYBL2* and *BjA07.MYBL2*, were expressed at significantly higher levels in apricot petals than in white petals (**Figure 5D**). The qPCR results were consistent with the transcriptomic data (**Figure 4A**). Collectively, functional annotation and independent expression validation strongly implicate *BjB03.PAP2* as the most promising candidate gene for the *APF* locus.

**Table 2:** Annotation of 23 upregulated DEGs screened from the BSA-seq intervals.

ID	Regulated	*Arabidopsis thaliana* ID	Description
*BjuOB03G35880*	Up	*AT1G02380*	Transmembrane protein
*BjuOB03G36400*	Up	*AT1G03000*	PEX6 (peroxin 6)
*BjuOB03G36920*	Up	*AT4G02930*	GTP binding Elongation factor Tu family protein
*BjuOB03G37500*	Up	*AT1G73370*	Encodes a protein with sucrose synthase activity (SUS6)
*BjuOB03G37810*	Up	*-*	Hypothetical protein Bca52824_015630 [*Brassica carinata*]
*BjuOB03G37950*	Up	*AT1G71695*	Peroxidase superfamily protein
*BjuOB03G37960*	Up	*AT2G36130*	Cyclophilin-like peptidyl-prolyl cis-trans isomerase family protein
*BjuOB03G38010*	Up	*AT1G78580*	Encodes an enzyme putatively involved in trehalose biosynthesis
*BjuOB03G38080*	Up	*AT5G24670*	ATTAD3, TAD3 (tRNA adenosine deaminase 3)
*BjuOB03G40770*	Up	*AT1G65590*	Encodes a protein with beta-hexosaminidase activity
*BjuOB03G41150*	Up	*AT1G66390*	MYB90, production of anthocyanin pigment 2 protein (PAP2)
*BjuOB03G42260*	Up	*AT1G67580*	CDKG2, cyclin-dependent kinase
*BjuOB03G43070*	Up	*AT5G18670*	BMY3, putative beta-amylase BMY3
*BjuOB03G43430*	Up	*AT1G68670*	HHO2, a homolog of HRS1; nitrate-inducible expression
*BjuOB03G44300*	Up	*AT1G69523*	S-adenosyl-L-methionine-dependent methyltransferases superfamily protein
*BjuOB03G44450*	Up	*AT1G66620*	Protein with RING/U-box and TRAF-like domain
*BjuOB03G44710*	Up	*AT2G02760*	Ubiquitin-conjugating enzyme UBC2; homolog of the yeast *RAD6* gene
*BjuOB03G44980*	Up	*AT1G70110*	Concanavalin A-like lectin protein kinase family protein
*BjuOB03G46340*	Up	*AT1G71380*	cellulase 3
*BjuOB03G46790*	Up	*AT5G52470*	FIB1 (FIBRILLARIN 1), encodes a fibrillarin
*BjuOB03G46800*	Up	*-*	Hypothetical protein F2Q70_00035224 [*Brassica cretica*]
*BjuOB03G47340*	Up	*AT4G28860*	Member of CKL gene family (CKL-A group)
*BjuOB03G48470*	Up	*AT1G73260*	Encodes a trypsin inhibitor involved in modulating programmed cell death in plant–pathogen interactions

We cloned and sequenced both the promoter and full-length genomic regions of this gene from the two parents to further investigate the molecular basis underlying the differential expression of the *BjB03.PAP2* gene between the parental lines CJ and WP. Sequence comparisons revealed no variation across the three exons; however, a single SNP (A-to-T) was identified in intron 1 (**Figure 5E**; **Supplementary Figure 4**). Because this SNP is also present in the yellow-flowered reference genome AU213, it is unlikely to be responsible for the observed differences in flower color (**Figure 5E**).

Further analysis of the approximately 1,463 bp promoter region (based on the AU213 reference) identified an A-to-T substitution at position −860 bp in the high-anthocyanin parent CJ (**Figure 5E**). In contrast, the non-anthocyanin parent, WP, retained the A allele at this site, suggesting that this polymorphism may influence gene expression (**Figure 5E**; **Supplementary Figure 5**). Moreover, an interesting structural variation was detected upstream of the *BjB03.PAP2* gene. At approximately −413 bp relative to the transcriptional start site in the reference genome, we identified a (TC)_n_ tandem repeat (**Figure 5E**). The reference genomes Tumida and AU213 carried an exceptionally long repeat of (TC)_84_ and (TC)_164_, respectively, whereas WP contained (TC)_76_ (**Figure 5E**). A parallel pattern was observed for the low-expression homolog *BjA07.PAP2*, in which the (TC)_n_ repeat length in its promoter region varied between 41 and 63 units across the four accessions (**Figure 5E**). Notably, in the high-anthocyanin parent, CJ, this repeat was substantially shorter and consisted only of (TC)_15_ (**Figure 5E**).

To investigate the evolutionary origin of these sequence variations, we conducted an analysis of the sequence features in the *PAP2* promoter region across diploid progenitor species of Brassica. First, we identified a large-scale deletion within the *PAP2* promoter region in all three available reference genomes of the diploid progenitor species *B. nigra* (**Supplementary Figure 5B**). This deletion spans the putative (TC)_n_ repeat region. Second, consistent presence of long (TC)_n_ repeats (64–74 units) was observed in the promoters of *PAP2* homologs across all three *B. rapa* reference genomes (**Supplementary Figure 5B**), which were derived from accessions exhibiting anthocyanin-deficient petals. Given that long tandem repeats in promoter regions are potent regulators of gene expression (typically repressive), this marked difference in repeat length may significantly contribute to the differential expression of *BjB03.PAP2* in CJ and WP.

## 4 Discussion

Petal coloration in Brassicaceae plants is primarily determined by the accumulation of two pigment classes, anthocyanins and carotenoids, or the absence of pigments, which results in white petals. For instance, anthocyanin accumulation produces blue, pink, purple, magenta, or violet−blue hues, as seen in purple-, pink-, white-, red-, or blue−flowered *Matthiola incana* (Chen et al., 2025), and pale purple−flowered *O. violaceus* also belongs to this group (Zhang et al., 2023). In contrast, yellow petals result from carotenoid accumulation, as exemplified by genera such as *Brassica*, *Isatis*, and *Barbarea*. In addition, some taxa are predominantly white-flowered, such as *Arabidopsis*, *Capsella*, and *Berteroa*. It is noteworthy that flower color is not always fixed within a given species or genus; some individuals may co-accumulate both pigment types, whereas others may lack pigments and appear white. Brassica crops are typically known for their yellow flowers. Many closely related species accumulate anthocyanins in their petals, implying that the ancestral lineage possesses the genetic potential for anthocyanin synthesis. Nevertheless, wild and native Brassica germplasms with anthocyanin-pigmented petals are scarce in both research and collections. In contrast, anthocyanin accumulation in vegetative tissues (e.g., leaves and stems) is more commonly documented across the genus. Notable examples include purple−leafed *B. juncea* regulated by *BjPur* or *BjMYB113* (Heng et al., 2020; Zhang et al., 2024; Cheng et al., 2026), *B. carinata* with purple leaves and stems regulated by *BcaB05.MYB114* (Chao et al., 2025), purple−leafed *B. napus* controlled by *BnaA.PL* (Li et al., 2016), as well as purple−leafed cabbage (*B. oleracea*) (Zhang et al., 2015b) and purple bok choy (*B. rapa*) (Zhang et al., 2014). The discovery of a wild *B. juncea* germplasm that accumulates anthocyanins in its petals represents a significant breakthrough (**Figure 1**). This finding compels a re-evaluation of the evolutionary trajectory of flower color in this species. It opens new avenues for elucidating the genetic basis and molecular regulatory mechanisms underlying this trait.

Studies on a resynthesized white−flowered *B. napus* line ‘2127’ revealed that its lack of yellow coloration results from the functional expression of *BnaC3.CCD4* located on the CC subgenome, degrading carotenoids and thereby eliminating yellow pigmentation (Zhang et al., 2015a). Further investigations have indicated that yellow-flowered accessions of *B. napus* (AACC), *B. carinata* (BBCC), and *B. oleracea* (CC) carry loss-of-function alleles of *CCD4* (Zhang et al., 2015a). These results collectively support the hypothesis that the white−petal trait likely represents the ancestral state in Brassica crops. In contrast, the widespread yellow−petal phenotype evolved through natural mutations disrupting *CCD4* function. Although the evolutionary transition from white to yellow petals via *CCD4* inactivation is well understood, the absence of anthocyanin-based pigmentation in cultivated Brassica species remains unknown. In this context, the anthocyanin-accumulating *B. juncea* germplasm CJ identified in this study provides important insights. Genetic analysis indicated that apricot-flowered traits were controlled by a single dominant *APF* locus (**Table 1**). The four flower colors observed in the F_2_ population reflect distinct patterns of carotenoid and anthocyanin accumulation. Specifically, the apricot phenotype results from the co-accumulation of both carotenoid and anthocyanin pigments. Pink petals are characterized by the presence of anthocyanins and a complete absence of carotenoids, whereas yellow flowers arise from carotenoid accumulation in the absence of anthocyanins. In contrast, the white phenotype is due to the lack of both pigment classes. Analogous to the case of *BnaC3.CCD4* in yellow flower evolution, the anthocyanin-based color in CJ may also represent an ancestral trait that was subsequently lost during domestication or evolution, potentially through mutations or promoter variations in key regulatory genes.

Based on the presence or absence of anthocyanins in petals, we performed BSA-seq by constructing extreme pools from an F_2_ population. This approach delimited the *APF* locus to a 9.76 Mb interval on chromosome B03 and contained 1,406 annotated genes (**Figure 3**). Within this interval, only two known genes involved in anthocyanin metabolism and regulation were identified: the positive regulator *BjB03.PAP2* and the negative regulator *BjB03.MYBL2* (**Figure 3**). Both genes encode classic components of the MBW transcriptional complex. *PAP2* belongs to the R2R3-MYB family of transcription factors. In *Arabidopsis*, it positively regulates anthocyanin biosynthesis, and overexpression of *AtPAP2* leads to anthocyanin accumulation in leaves and light purple to red flowers (Borevitz et al., 2000). Similarly, in *B. napus*, overexpression of *PAP2* from *O. violaceus* or endogenous *BnaA07.PAP2* can induce anthocyanin accumulation, resulting in red or apricot-colored petals (Fu et al., 2018; Ye et al., 2022). In contrast, the MBW complex activity is repressed by *MYBL2*; mutations in *MYBL2* lead to anthocyanin accumulation in *Arabidopsis*, highlighting its role as a key negative regulator (Dubos et al., 2008; Xie et al., 2016). We conducted a comparative transcriptome analysis of floral buds at four developmental stages from the anthocyanin-accumulating line, CJ, to the non-accumulating line, WP, to rapidly assess gene expression within the BSA candidate interval. Our analysis revealed that LBGs of the anthocyanin pathway, such as *FLS*, *DFR*, *ANS*, *UFGT*, *MT*, and *GST*, were significantly upregulated in CJ buds (**Figure 4A**). Notably, *BjB03.PAP2* was consistently upregulated across all four developmental stages in CJ (**Figure 4B**). We hypothesized that the elevated expression of *BjB03.PAP2* activates the MBW transcriptional complex, thereby leading to the upregulation of anthocyanin LBGs. In contrast, the negative regulator *BjB03.MYBL2* showed upregulated expression in CJ at stages S3 and S4 (**Figure 4A**). Theoretically, this would suppress MBW complex activity and reduce anthocyanin production. Considering this inconsistency, along with the fact that the *APF* locus is a single dominant gene, *B03.MYBL2* was promptly excluded from the list of candidate genes.

As the most critical candidate gene, *BjB03.PAP2* was analyzed for its relative expression level in flowers of four different colors from the F_2_ population (**Figure 5A**). Its expression was significantly upregulated in the anthocyanin-accumulating petals, which was consistent with the transcriptomic data (**Figure 4A**). Comparative sequencing of the coding region revealed no polymorphisms among the four materials tested, including the two parental lines (CJ and WP) and two reference genomes (**Figure 5E**). Given that promoter variations critically regulate *PAP2* expression, we focused on the promoter region. This is supported by prior findings in *B. napus*, where the co-insertion of a 412- and 210-bp fragment upstream of *BnaA07.PAP2*, presumed to be an enhancer element, enhances its expression and activate the anthocyanin biosynthesis pathway (Ye et al., 2022).

This study revealed an extreme length polymorphism of the (TC)_n_ repeat in the *BjB03.PAP2* promoter, ranging from satellite DNA-like (TC)_164_ (in reference AU213) to microsatellite-like (TC)_15_ (**Figure 5E**). This variation likely arose from the slippage of DNA replication. Analogous regulatory mechanisms involving dinucleotide repeat polymorphisms have been reported for other systems. For example, expression of the human *HMGA2* promoter is co-regulated by a polymorphic (TC) repeat, underscoring the conserved role of such simple sequence repeats in fine-tuning gene expression across species (Borrmann et al., 2003). Similarly, in potatoes, the natural variation in the (TA)_n_ repeat number (TA_10_ vs. TA_13_) within the promoter of the *SGT3* gene differentially regulates promoter activity, thereby influencing steroidal glycoalkaloid biosynthesis (Zhou et al., 2025). Given that long TC repeats can form Z-DNA structures and strongly repress transcription, we propose that this *APF* locus has undergone key regulatory evolution: in the WP and reference genome lineages, repeat expansion created an effective silencing element, which may have been selected to suppress gene expression, whereas in CJ, the shorter repeat retains the ancestral potential for high expression. Thus, this tandem repeat may act as a rapidly evolving “tuner” that fine−tunes flower color adaptation in this species.

Future studies should focus on three key directions to elucidate the role of *BjB03.PAP2* and its promoter variation: (1) direct functional validation of *BjB03.PAP2* via transgenic overexpression and gene editing; (2) systematic haplotype analysis of the *BjB03.PAP2* promoter, particularly the (TC)_n_ repeats, across diverse *B. juncea* germplasms to uncover its population genetic basis; and (3) transgenic complementation assays comparing promoters with different (TC)_n_ lengths to confirm their cis-regulatory roles *in vivo*. Together, these approaches will clarify how this “molecular tuner” drives adaptive evolution of flower color in rapeseed.

## Data Availability

*Brassica juncea* gene sequence data can be accessed from the BRAD database. The raw data of RNA seq and BSA-seq is deposited in National Genomics Data Center (NGDC) under the Bioproject PRJCA054130.

## Supplementary material

Supplementary Figure 1 Flower color phenotypes and anthocyanin accumulation in F_1_ and F_2_ populations at different developmental stages.

Supplementary Figure 2 Sequencing statistics and genome-wide single-nucleotide polymorphism (SNP) distribution for bulked segregant analysis.

Supplementary Figure 3 Correlation and principal component analyses of RNA-seq samples from CJ and WP flower buds at different developmental stages.

Supplementary Figure 4 Sequence variations in the *BjB03.PAP2* gene between the parental lines CJ and WP.

Supplementary Figure 5 Structural variations in the promoter region of *PAP2* between different accessions.

Supplementary Table 1 Primer sequences used for qRT-PCR and sequencing analyses.

Supplementary Table 2 Analysis of differential anthocyanin profiles in flower buds of CJ and WP across the four developmental stages.

Supplementary Table 3 Summary of read duplication statistics from the BSA-seq analysis.

Supplementary Table 4 Statistics of the RNA-seq data alignment efficiency to the reference genome.

Supplementary Table 5 Statistics of differential gene expression in the anthocyanin metabolic pathway across the four bud developmental stages in CJ and WP.

Supplementary Table 6 Statistics of differential expression for candidate genes within the 9.76-Mb interval identified using BSA.

Supplementary Table 7 Candidate genes within the BSA-defined interval that were consistently differentially expressed across all the four bud developmental stages in CJ and WP.
