# Supplementary material for: Beyond yellow: discovery and genetic dissection of an apricot petal color in Brassica juncea L
Source: Front Plant Sci. 2026 Feb 2;17:1767871. doi: 10.3389/fpls.2026.1767871 (PMC12907188; doi:10.3389/fpls.2026.1767871)
Supplement: Supplementary Figure 1 — Flower color phenotypes and anthocyanin accumulation in F1 and F2 populations at different developmental stages. [file DataSheet1.docx]

Supplementary Material

# Supplementary Figures and Tables


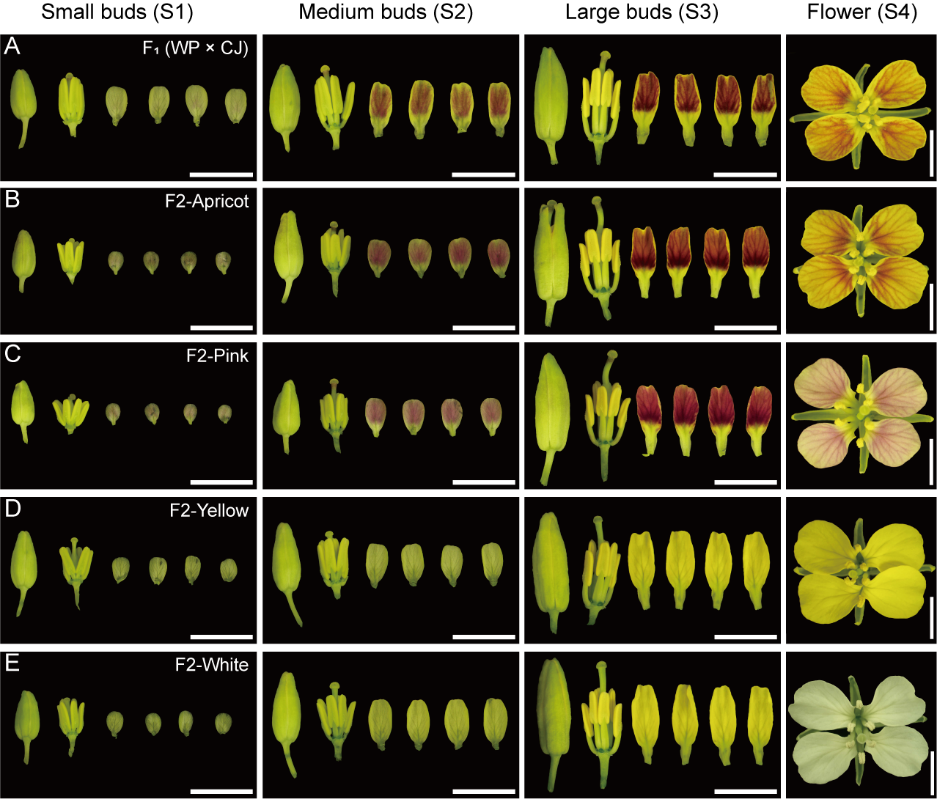


**Supplementary Figure 1.** Flower color phenotypes and anthocyanin accumulation in F_1_ and F_2_ populations at different developmental stages. (A) Flower buds and fully opened flowers of F_1_ plants. (B–D) Flower buds and fully opened flowers representing four distinct color phenotypes segregating in the F_2_ population. Scale bar: 5 mm.


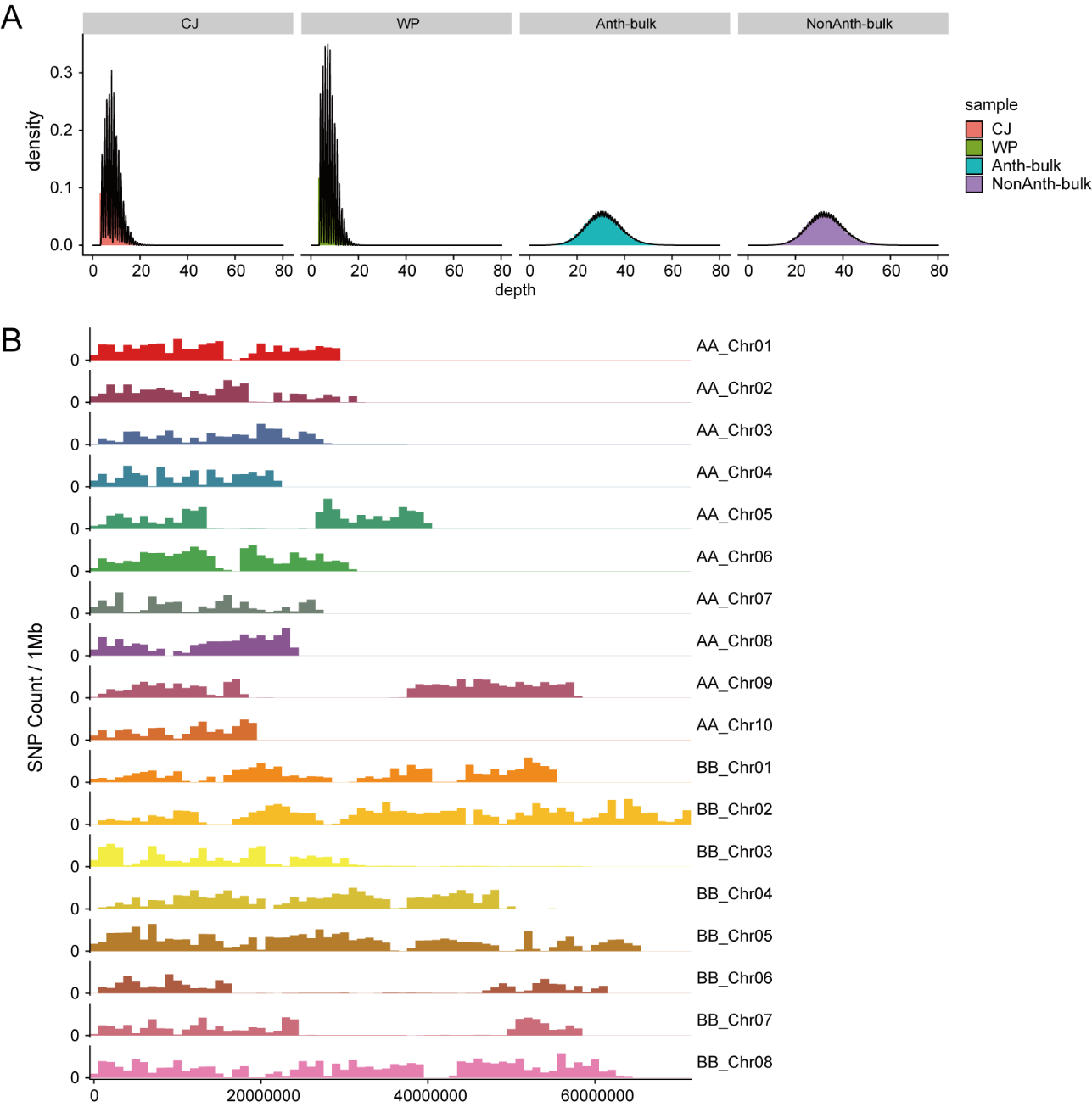


**Supplementary Figure 2.** Sequencing statistics and genome-wide single-nucleotide polymorphism (SNP) distribution for bulked segregant analysis. (A) Sequencing depth distribution for the parental lines and phenotype-based DNA bulks. (B) Distribution density of SNPs across the 18 chromosomes of *Brassica juncea*.


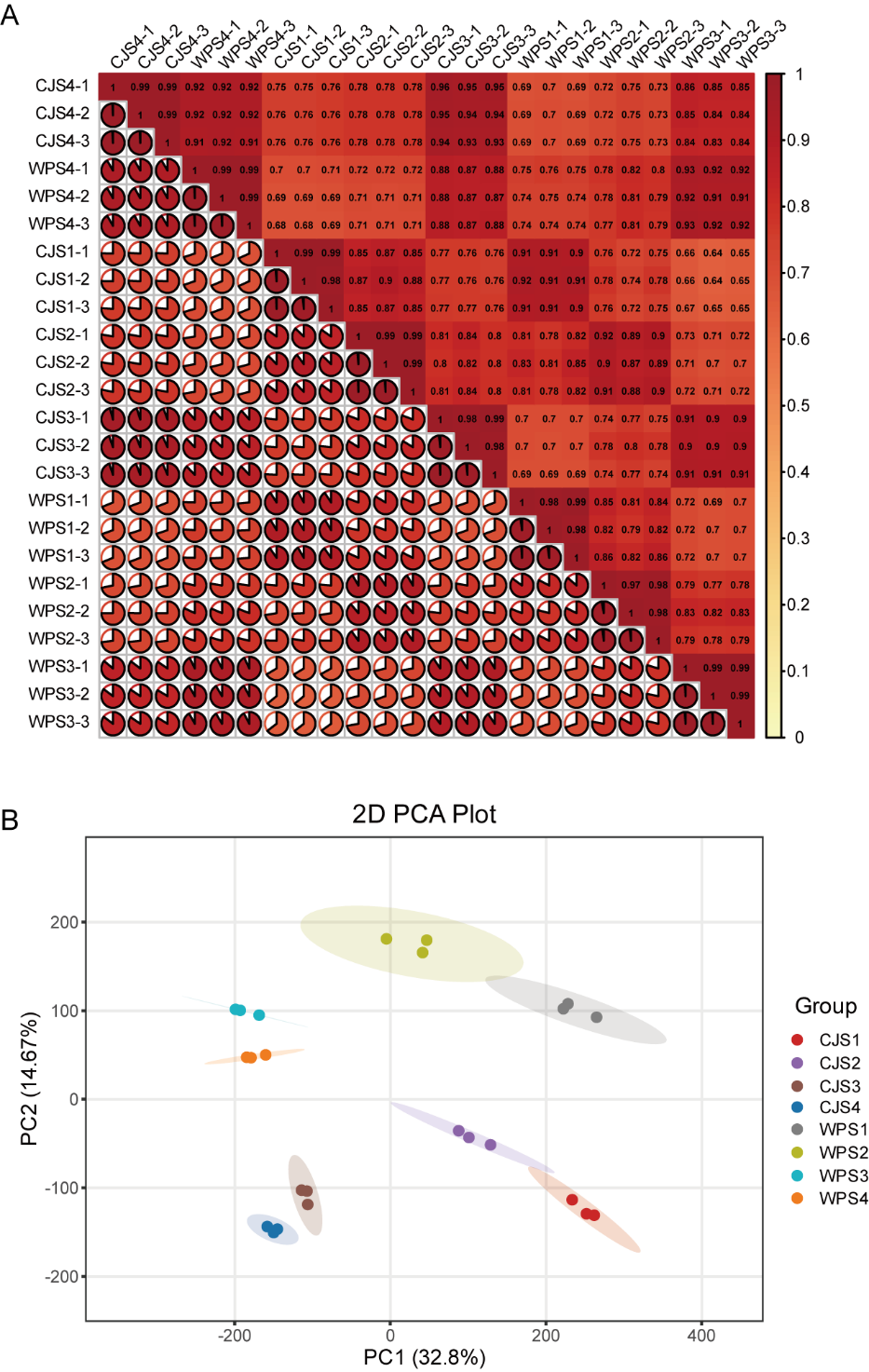


**Supplementary Figure 3.** Correlation and principal component analyses of RNA-seq samples from CJ and WP flower buds at different developmental stages. (A) Correlation matrix of gene expression profiles across 24 RNA-seq samples. (B) PCA plots of all samples based on the global gene expression patterns.


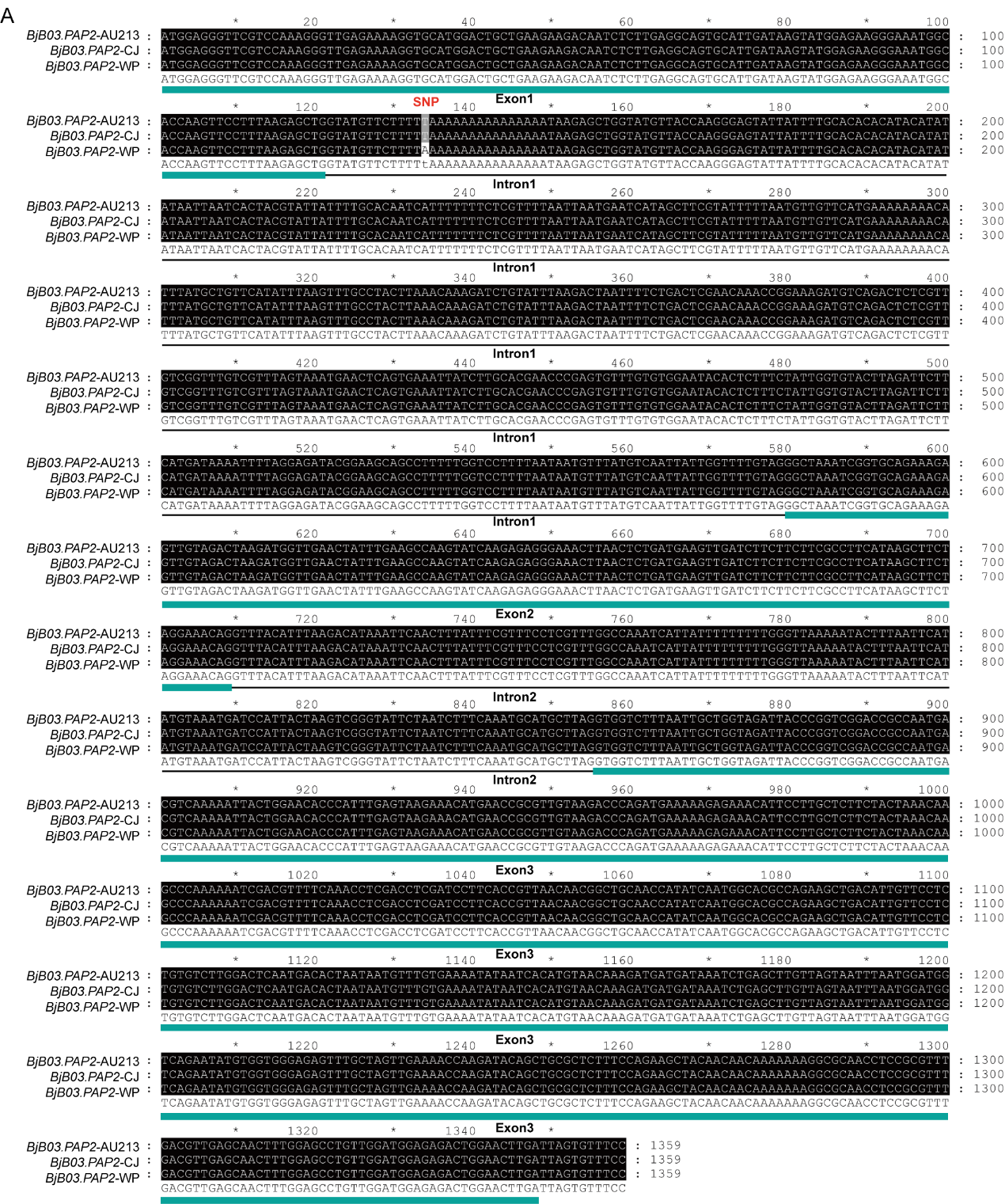


**Supplementary Figure 4.** Sequence variations in the *BjB03.PAP2* gene between the parental lines CJ and WP. (A) Schematic representation of the full-length *BjB03.PAP2* gene structure, from the start to the stop codon. Sequence variations identified between CJ and WP are highlighted in red.


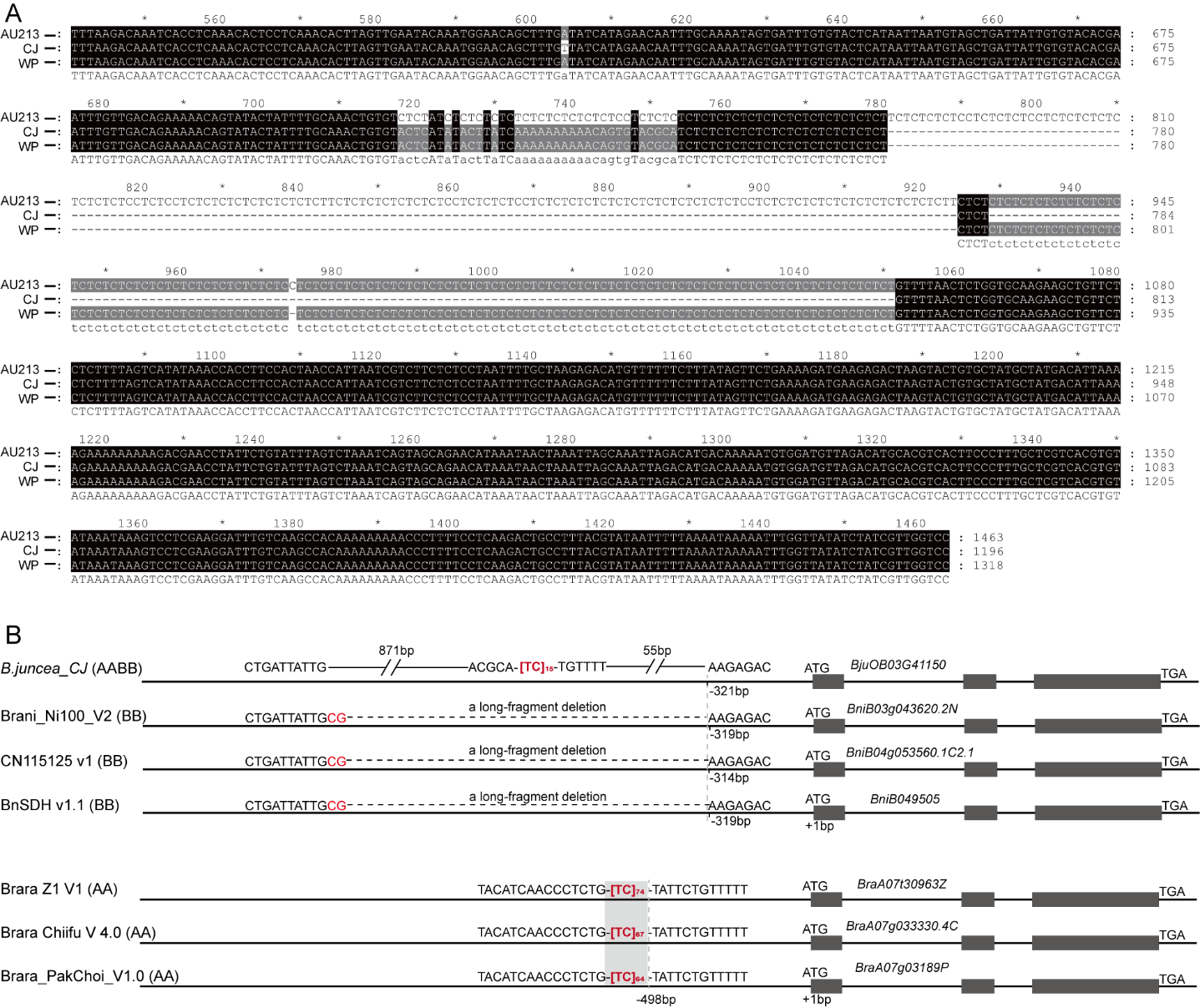


**Supplementary Figure 5.** Structural variations in the promoter region of *PAP2* between different accessions. (A) Detailed view of the 923-bp promoter sequence upstream of the *BjB03.PAP2* start codon, based on the AU213 reference genome. (b) Comparative sequence analysis of the *PAP2* promoter homologs among different diploid progenitor species, *B. nigra* and *B. rapa*.

**Supplementary Table 1:** Primer sequences used for qRT-PCR and sequencing analyses.

**Supplementary Table 2:** Analysis of differential anthocyanin profiles in flower buds of CJ and WP across the four developmental stages.

**Supplementary Table 3:** Summary of read duplication statistics from the BSA-seq analysis.

**Supplementary Table 4:** Statistics of the RNA-seq data alignment efficiency to the reference genome.

**Supplementary Table 5:** Statistics of differential gene expression in the anthocyanin metabolic pathway across the four bud developmental stages in CJ and WP.

**Supplementary Table 6:** Statistics of differential expression for candidate genes within the 9.76-Mb interval identified using BSA.

**Supplementary Table 7:** Candidate genes within the BSA-defined interval that were consistently differentially expressed across all the four bud developmental stages in CJ and WP.
